# Frequency and factors associated of potential zoonotic pathogens (*Borrelia* spp., *Rickettsia* spp., *Leishmania* spp., and *Anaplasma phagocytophilum*) in equids in the state of Bahia, Brazil

**DOI:** 10.1186/s13071-021-04777-4

**Published:** 2021-05-22

**Authors:** Sonia Carmen Lopo Costa, Jéssica de Souza Freitas, Fábio Santos Carvalho, Maria Julia Salim Pereira, Matheus Dias Cordeiro, Adivaldo Henrique da Fonseca, Márcia Mariza Gomes Jusi, Rosangela Zacarias Machado, Alexandre Dias Munhoz

**Affiliations:** 1 Department of Agricultural and Environmental Sciences, State University of Santa Cruz – UESC, Soane Nazaré de Andrade Campus, Ilhéus, BA Brazil; 2 Department of Animal Parasitology, Rural Federal University of Rio de Janeiro-UFRRJ, Seropédica, RJ Brazil; 3 Department of Epidemiology and Public Health, Rural Federal University of Rio de Janeiro-UFRRJ, Seropédica, RJ Brazil; 4 Department of Animal Pathology, Julio Mesquita Filho State University, UNESP/Jaboticabal, Rod. Paulo Castelanne s/n, Jaboticabal, São Paulo, 4884-900 Brazil

**Keywords:** Zoonoses, Lyme disease, Spotted fever, Leishmaniasis, Horses, Donkeys, Mules

## Abstract

**Background:**

Currently, various zoonotic diseases are classified as emerging or reemerging. Because equids have a direct relationship with various vectors, they are possibly more frequently exposed to zoonotic agents than are humans. The undeniable importance of diseases such as human granulocytic anaplasmosis, spotted fever, and leishmaniasis for both public and animal health, as well as the possibility of equids acting as sources, reservoirs, or even sentinels for these pathogens, justifies the detection of their frequency and factors associated with infection in equids from northeastern Brazil.

**Methods:**

Blood samples were collected from 569 equids (528 horses, 33 donkeys, and 8 mules), 516 from a rural area and 53 from an urban area. Pathogen detection was carried out as follows: *Borrelia* spp. and *Rickettsia* spp., serological analysis; *Leishmania* spp., serological analysis and polymerase chain reaction (PCR); *Anaplasma phagocytophilum,* PCR. Determination of associated factors was carried out through generalized linear models*.*

**Results:**

The frequencies of positivity for the pathogens observed in equids were as follows: *Borrelia* spp., 13.9% (79/569); *Leishmania* spp., 3.5% (20/569); *Rickettsia* spp. 33.4% (190/569). Regarding factors associated with infection, male sex was associated with protection against *Borrelia spp.*; donkeys and mules were associated with protection against *Rickettsia* spp., while a younger age was a risk factor. The infection of *A. phagocytophilum* was not detected in the sampled population. Co-infection was detected in 5.1% (29/569) of the animals.

**Conclusions:**

Most of the studied pathogenic agents are present in the prospected area, indicating a possible risk for both human and animal health. This demonstrates that equids can be considered important sentinels in the assessment of pathogens with zoonotic potential in the region.

**Graphical Abstract:**

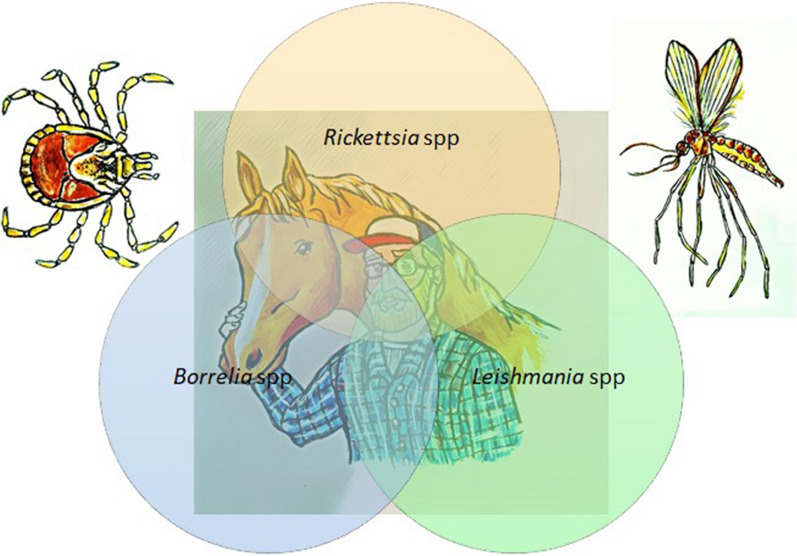

**Supplementary Information:**

The online version contains supplementary material available at 10.1186/s13071-021-04777-4.

## Background

Equids can be infected with various pathogens that also infect humans [1–3]. The close contact between these two species, which spans for over 5000 years, potentially allowed some agents to develop the capacity to develop and/or multiply in both of them. In studies on populations, concepts like ecological fitting and fitness space, which explain the capacity for interactions among microbiota, flora, fauna, climate, and environment in the search for equilibrium, also seem to explain this phenomenon [4].

Currently, various zoonotic diseases are classified as emerging or reemerging [5]. Climate change, increased possibility of dispersion, and interactions between vertebrate and invertebrate hosts, seem to be associated with the appearance, or even outbreaks, of certain diseases in different regions of the planet [4, 6, 7].

Because equids have a direct relationship with various vectors, which is associated with the environment and management practices they are exposed to, they can be more frequently exposed to hemoparasites and other zoonotic agents than are humans [8–10]. As such, they are able to play an important role as sentinels for certain diseases [11–15].

Diseases such as human granulocytic anaplasmosis, spotted fever, and leishmaniasis have an impact on public health. This causes significant direct and indirect economic losses, morbidity, and, in some cases, death in the human population. Similar to leishmaniasis [16], these diseases are highly prevalent in certain regions of the world. The causative agents of these illnesses also infect equids and invertebrate hosts present in the same environment, potentially increasing their exposure to infection. Horses are normally carriers, but clinical signs of borreliosis (Lyme disease) [17, 18], granulocytic anaplasmosis [19, 20], leishmaniasis [21, 22], and rickettsiosis [23, 24] have been described in previous reports.

The undeniable importance of the above-mentioned diseases for public and animal health, as well as the possibility of equids acting as sources, reservoirs, or even sentinels for these pathogens, justifies the detection of the frequency and factors associated with infections in equids from northeastern Brazil.

## Methods

### Study area and sample population

Data were collected between August 2013 and December 2014 in the microregion of Ilhéus-Itabuna, in the state of Bahia, northeastern Brazil. This geographic region is part of the mesoregion of southern Bahia and has an estimated equid population of 90,974 animals [25]. The study area is located in the Atlantic Forest. The annual average rainfall is 1445 mm, with a relative humidity of 80% and a temperature of 24 °C [26]. Five counties from this mainly rural microregion were selected for the study and were ranked according to the size of their equid population as follows: Itaju do Colônia (15° 08′ S 39° 43′ O), Itapé (14° 52′ S 39° 25′ O), Ibicaraí (14° 51′ S 39° 35′ O), Santa Cruz da Vitória (14º57'S 39° 48′ O), and Floresta Azul (14° 50′ S 39° 39′ O). The county of Itabuna (15° 8′ S 39° 43′ W), which is a mostly urban area, was also included in the study (Additional file 1: Figure S1).

Animals, farms, and counties were selected based on their convenience. The number of animals per county was proportional to their equid population. Blood samples were collected from 569 equids (528 horses, 33 donkeys, and 8 mules); 516 out of 569 equids were from 20 rural properties; 53 horses out of 569 equids were from urban areas and were used by mounted police, to draw coaches, or for horseback riding.

To evaluate potential risk factors, information regarding signalment (species, age, sex), farm characteristics, and management (animal kept in a stable, presence of ticks, contact with other animal species) was obtained through semi-structured interviews with handlers (staff) or owners. Interviews were always conducted by the same researcher.

The study was carried out according to the standards established by the Brazilian College of Ethics and Animal Welfare. The research proposal was approved by the Committee for Ethics in Research with Animals (protocol 002/2013) at the State University of Santa Cruz, Ilhéus, BA, Brazil.

### Sample collection and processing

Blood (20 ml) was collected from each animal through jugular venipuncture using disposable needles (25 × 8 mm) connected to vacuum tubes with and without anticoagulant (EDTA). Tubes with anticoagulant were centrifuged for 10 min at 699×*g*. The supernatant plasma was discarded and then both the leukocyte layer and the packed red blood cells were removed, poured into DNase- and RNase-free plastic tubes, and frozen at − 20 °C for subsequent DNA extraction. To obtain the sera, the tubes without anticoagulant were centrifuged at 699×*g* for 10 min; the sera separated through aspiration were placed in plastic tubes and frozen at − 20 °C until the serological tests were performed.

Ticks were collected from equids during visits to farms. Taxonomic identification of the ticks was performed using a stereoscopic microscope, according to previously published morphological keys [27–29]. The ticks were stored in micro-tubes or Falcon tubes, depending on the quantity, and labeled with the name of the property from which they were collected. Subsequently, they were submerged in absolute alcohol and stored at − 20 °C for future morphological identification and DNA extraction.

### Serology

#### *Borrelia* spp.

For the detection of antibodies against the crude *B. burgdorferi* (*s.l.*) antigen G39/40 strain, indirect ELISA was performed using the antigen at a concentration of 15 µg/ml, serum in phosphate buffer saline (PBS Tween 20, 0.05%, pH 7.4) at a dilution of 1:800, and conjugated antibodies at a dilution of 1:5000 (anti-horse IgG, A6063, Sigma®) [30]. The cut-off point for the assay was determined using the previously described methodology [31], which is 2.5 times the mean value of negative control (animal previously tested) absorbance. Optical density was measured at 405 nm. Four negative samples from previously tested animals were used as negative controls, and there were two positive controls from animals inoculated with crude *B. burgdorferi* antigen, G39/40 strain.

#### *Leishmania* spp.

For the detection of antibodies against the soluble *L. braziliensis* antigen, indirect ELISA was performed using the antigen at a concentration of 10 ng/ml, serum in phosphate buffer saline (PBS Tween 80, 0.05%, pH 7.4) at a dilution of 1:200, and conjugated antibodies at a dilution of 1:30,000 (anti-horse IgG, A6063, Sigma®) [31]. The cut-off point for the assay was determined using the previously described methodology [31], which is 2.5 times the mean value of negative control (animal previously tested) absorbance. Optical density was measured at 405 nm.

#### *Rickettsia rickettsii* and *R. parkeri*

The sera were tested using an indirect fluorescent antibody test (IFAT); a dilution of 1:64 [32] was the cut-off point for antigens of *R. rickettsii* Taiaçu strain [33] and *R. parkeri* At24 strain [34]. The slides had been previously sensitized with an antigen produced through the cultivation of *R. rickettsii* and *R. parkeri* in Vero cells. Conjugated Anti-Horse IgGs (F7759, Sigma-Aldrich ®) were used at a dilution of 1:80 [32]. A microscope with an epifluorescence system (OLYMPUS, BX 51) was used for reading the slides. Reactions with complete fluorescence in the periphery of the agents were considered positive. Positive and negative controls were obtained from the Molecular Epidemiology Laboratory of the Fluminense Federal University (UFF). The positive samples were two-fold titrated.

### PCR

#### Extraction of DNA from equine blood samples and molecular diagnosis

DNA was extracted from blood samples using a commercial QIAamp® DNA Blood Mini Kit (QIAGEN™), according to the manufacturer's recommendations. DNA samples were labeled with accession numbers and stored in a freezer at − 20 °C for subsequent polymerase chain reaction (PCR).

#### *Leishmania* spp.

Specific RV1/RV2 primers (Table 1) for *L. infantum* [35] and B1/B2 primers (Table 1) for *L. braziliensis* were used [36]*.* PCR was carried out using 10 × Taq DNA polymerase buffer, 0.2 mM of MgCl_2_, 2 mM of each dNTP, 10 pmol of each *L. infantum* primer, 1.25 U of Taq DNA polymerase (Invitrogen®), and 5 µl of genomic DNA. Similar conditions were adopted for *L. braziliensis* using 3 mM of MgCl_2_ and 2.5 U of Taq DNA polymerase. The thermocyclic conditions were as follows: initial denaturation, 94 °C for 5 min; 35 cycles of denaturation, 94 °C for 1 min, annealing of primers, 59 °C (*L. infantum*) or 65 ºC (*L. braziliensis*) for 1 min and extension, 72 ºC for 1 min; final extension, 72 °C for 5 min. Ultrapure water was used as a negative control. Positive controls of the Huhu strain were provided by the Gonçalves Muniz Research Center (FIOCRUZ-BA).

**Table 1:** Primers used for PCR with respect to *Leishmania infantum, L. braziliensis*, *Anaplasma phagocytophilum, Rickettsia* spp., ticks (16S rRNA) and GAPDH

Target	Code	Sequence of oligonucleotides (5′–3′)	Reaction	Reference
*Leishmania infantum*	RV1	CTTTTCTGGTCCCGCGGGTAG	1ª	Lachaud et al. [35]
	RV2	CCACCTGGCCTATTTTACACCA		
*Leishmania braziliensis*	B1	GGGGTTGGTGTAATATAGTGG	1ª	De Bruijn and Barker [36]
	B2	CTAATTGTGCACGGGGAGG		
*Anaplasma phagocytophilum*	gE3a	CACATGCAAGTCGAACGGATTATTC	1ª	Massung et al. [37]
	gE10R	TTCCGTTAAGAAGGATCTAATCTCC		
	gE2	GGCAGTATTAAAAGCAGCTCCAGG	2ª	
	gE9f	AACGGATTATTCTTTATAGCTTGCT		
GAPDH*	gapF	CCTTCATTGACCTCAACTACAT	1ª	Birkenheuer et al. [38]
	gapR	CCAAAGTTGTCATGGATGACC		
*Ticks (16S rRNA)*	–	CCGGTCTGAACTCAGATCAAG	1ª	
	–	GCTCAATGATTTTTTAAATTGCTGT		Mangold et al. [41]
*Rickettsia* spp	CS 239	GCTCTTCTCATCCTATGGCTATTAT	1ª	
	CS1069	CAGGGTCTTCGTGCATTTCTT		McIntosh et al. [42]

^*^Glyceraldehyde-3-phosphate dehydrogenase

#### *Anaplasma phagocytophilum*

Reactions were carried out using primers described in a previous study [37] to increase the region of the 16S rRNA gene, gE3a/gE10R in the first reaction (932pb), and gE2/gE9f in the second reaction (546pb) (Table 1), using a final volume of 12.5 µl containing 2.5 µl of genomic DNA, 10 × reaction buffer, 1.5 mM of MgCl_2_, 0.2 mM of each dNTP, 0.4 µM of each primer, and 1.25 U of Taq polymerase; ultrapure water was added until the final volume was obtained. The thermocyclic conditions were as follows: initial denaturation, 94 °C for 5 min; 40 cycles of denaturation, 94 °C for 30 s, annealing, 55 °C for 1 min, extension, 72 °C for 1 min; and final extension, 72 °C for 5 min [37]. For the nested-PCR, the same concentrations and final volume were used for the “mix,” that is, 0.5 µl of the first reaction’s product was used. The number of cycles was reduced to 30 while maintaining the thermocycler’s time and temperature conditions. Ultrapure water was used as a negative control.

#### PCR for GAPDH (glyceraldehyde-3-phosphate dehydrogenase) detection

To verify DNA integrity and the presence of potential inhibitors, negative samples were subjected to PCR for detection of the GAPDH gene using primers described by Birkenheuer et al. [38] (Table 1). In the PCR reactions, a final volume of 25 μl was used, composed of 5 μl of genomic DNA, 10 × reaction buffer, 2.0 mM of MgCl_2_, 0.2 mM of each dNTP, 0.4 μM of each primer, 1.25 U of Taq polymerase, and ultrapure water until the final volume was reached. The amplification protocol used consisted of an initial denaturation step at 95 °C for 5 min, followed by 40 cycles at 94 °C for 30 s for denaturation, annealing at 52 °C for 1 min, extension at 72 °C for 1 min, and final extension at 72 °C for 5 min [39].

#### Extraction of genomic DNA from the ticks and PCR for *Rickettsia* spp. detection

Total DNA was extracted from the ticks individually, using the phenol–chloroform method [40].

All the samples were subjected to a reaction using specific oligonucleotides for the *16S* gene (Table 1) to verify DNA integrity and the possible presence of PCR inhibitors. The thermocyclical conditions used were as follows: initial denaturation, 94 °C for 2 min; 35 cycles of denaturation, 94 °C for 30 s, annealing, 55 °C for 30 s, extension, 72 °C for 45 s; the final extension, 72 °C for 7 min [41].

To determine the presence of *Rickettsia* spp. DNA, the pair of primers CS-239 and CS-1069 was used, which amplifies 834 pb of the *gltA* gene (Table 1). This gene is present in all known *Rickettsia* species. The thermocyclical conditions used were as follows: initial denaturation, 95 °C for 5 min; 40 cycles of denaturation, 95 °C for 20 s, annealing, 52 °C for 20 s, extension, 72 °C for 40 s; final extension, 72 °C for 5 min [42].

PCR products were detected using 2% agarose gel electrophoresis in a tris-acetate-EDTA (TAE) running buffer (40 mM Tris-acetate, 2 mM EDTA pH 8.0). The gel was run at 80 V, 180 mA for 30 min, and then stained with ethidium bromide (0.5 μg/ml). A DNA molecular weight standard control (1 Kb Plus DNA Ladder, Invitrogen®) was used to estimate the size of the amplified products. Amplified products were visualized under ultraviolet (UV) transilluminator (LPIX, Loccus Biotecnologia®) and photographed on a coupled image analyzer.

### Statistical analysis

The variables were categorized for the purposes of statistical modeling as follows: species (horse or donkey + mule); age, in the form of age ranges, i.e. young (≤ 3 years), adult (> 3 and < 12 years), or senior (≥ 12 years); sex (male or female); equids kept in a stable (yes or no); equids who had contact with sheep (yes or no); equids who had contact with goats (yes or no); equids who had contact with poultry (yes or no); equids who had contact with cattle (yes or no); presence of rats on the farm (yes or no); presence of toxic plants in the pastures (yes or no); and inserted into the models according to the biological plausibility for each agent. The presence of *Borrelia* spp., *Rickettsia* spp., *Leishmania* spp. and *Anaplasma phagocytophilum* infection (yes/no) was considered as outcome variables. Donkeys and mules were inserted into a single category because of the low number of animals.

Blood samples from animals from urban areas were collected only in the county of Itabuna; a total of 53 horses were sampled, including those used by the mounted police, to draw coaches, or for horseback riding. These animals were excluded from modeling because of the different management practices applied to these horses. They were only used to compare frequency distributions of agents between animals living in rural and urban areas.

Generalized linear models with binomial distribution were used to perform bivariable and multivariable analyses. Because of the possibility of clusters, intraclass correlation coefficients (ICCs) were calculated. Null models [43] were estimated to obtain ICCs for municipality and farm variables because of the possibility of observations of animals from the same municipality and/or farm being correlated, forming clusters [44]. In cases of cluster formation, the variables tested were considered random, and generalized linear mixed models were used for the analyses.

The modeling strategy used in the multivariable analyses was backward, that is, all variables were initially included in the model. Starting from this initial model, variables were selected at each step based on the Wald test until the most parsimonious model that best explained the outcome was obtained. The significance level for variables to remain in the final model was set at 5%. The Akaike information criterion (AIC) was used to evaluate the fit of the models. Frequency distributions among animals living in rural areas and animals living in urban areas were compared using the chi-square test.

Odds ratios (OR) and their respective 95% confidence intervals (CI) were calculated based on the regression coefficients that had been estimated through the models. Statistical calculations were performed using R software, version 3.2.5, for Windows [45] through the lmer4 package [46], version 1.1-12.

## Results

Regarding *Rickettsia* spp., 33.39% (190/569) of the equids showed positivity for at least one of the tested antigens. The proportion of animals that reacted serologically to only *R. rickettsii* antigens was 14.6% (83/569)—14.9% (77/516), 11.3% (6/53) of which belonged to the rural and urban populations, respectively (*p* > 0.05). The antibody titers varied from 1:64 to 1:1024 (Additional file 1: Table S1). Serologically reactive equids were found in all the studied municipalities, with the proportion of positivity varying from 6.6–44.7%. Among the 20 rural properties in the state, animals in five properties did not show positivity, while the proportion of positivity varied from 4.0–50.0% in the others. Positivity among the equids was distributed as follows: horses, 15.3% (81/528); donkeys and mules, 4.8% (2/41).

Regarding *R. parkeri* antigens, 15.1% (86/569) of the animals had antibodies against only these antigens—15.5% (80/516) and 11.3% (6/53) of which belong to the rural and urban populations, respectively (*p* > 0.05). The antibody titers varied from 1:64 to 1:512 (Additional file 1: Table S1). Positivity of equids was observed in all of the studied municipalities, with proportions varying from 5.6 to 33.7% (Additional file 1: Table S2). Among the 20 rural properties, animals in three properties did not show positivity. On the properties showing reactivity, the proportion of positivity varied from 3.3 to 31.5%. Among the evaluated species, positivity was distributed as follows: horses, 15.3% (81/528); donkeys and mules, 12.2% (5/41).

In 3.7% (21/569) of the animals, responses to both *R.* *parkeri* and *R. rickettsii* antigens were noted. Such animals constituted the “double-reaction” group. Among the animals presenting double reactivity, 66.6% (14/21) and 9.5% (2/21) had titers of 1:64 and 1:128, respectively, for both agents; however, five animals had divergent titers among the species.

The ICCs (intraclass correlation coefficients) for the municipalities (1.26%) and farms (2.96%) did not indicate the formation of clusters. Generalized linear models were therefore used (Additional file 1: Tables S3 and S4). The final model (Table 2) shows other species (donkey + mule) associated with protection and the young age group as a risk factor for contracting the infection.

**Table 2:** Generalized linear classic multivariate model for factors associated with *Rickettsia* spp. infections in equids. Final model

Variable	Category	Odds ratio (95% CI)	*p*
Species	Horses (Ref)		
	Donkey or mule	0.39 (0.17–0.91)	0.03
Age range	Young	2.01 (1.18–3.42)	0.01
	Adult	1.10 (0.72–1.65)	0.70
	Senior (Ref)		

AIC = 656.52 (Akaike information criterion)

The serological analysis for *Borrelia* spp. showed that 13.9% (79/569) of the animals tested positive, with 13.9% (72/516) and 13.2% (7/53) in the rural and urban zones (*p* > 0.05). Animals that tested positive were identified in all of the municipalities. On 85% (17/20) of the evaluated farms, at least one positive equid was found. Besides, 13.4% (71/528) of the horses and 19.5% (8/41) of the donkeys and mules tested positive for *Borrelia* spp. The ICCs for the counties (1.5%) and farms (4.5%) indicated clusters only at the farm level. Therefore, mixed regression models with a farm as the random effect were used for the analysis of possible risk factors for the occurrence of *Borrelia* spp. infection (Additional file 1: Table S5, S6) in the final model; this analysis indicated that male sex [OR 0.42(0.19–0.91); *p* = 0.03] was associated with protection against *Borrelia spp.*

For *Leishmania* spp., 3.5% (20/569) of animals tested seropositive in ELISA, all of which belonged to the rural zone; none of the animal tested positive as determined by PCR analysis. All the municipalities of the prospected rural area presented seropositive animals. At least one positive animal was observed on 40% (8/20) of the farms. Among the horses, 2.6% (14/528) tested positive for *Leishmania* spp., while this percentage was 14.6% (6/41) for donkeys and mules. Considering the low number of positive animals, which could generate erroneous results, performing a descriptive analysis of the results was considered appropriate. Thus, greater positivity was identified in donkeys and mules (Additional file 1: Table S7).

**Fig. 1:**
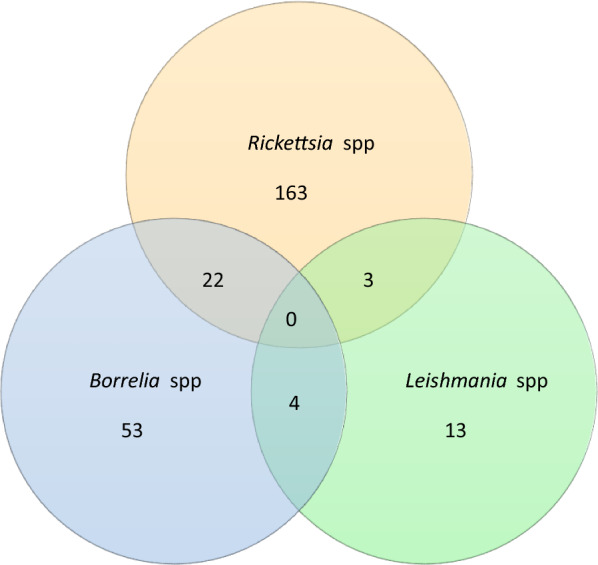
Distribution of infections and co-infections by *Borrelia* spp., *Rickettsia* spp., and *Leishmania* spp. in naturally infected equids in the lhéus-Itabuna microregion, Bahia

Distribution of co-infections among equids was as follows: *Borrelia* spp. and *Rickettsia* spp., 3.86% (22/569); *Borrelia* spp. and *Leishmania* spp., 0.7% (4/569); and *Rickettsia* spp. and *Leishmania* spp., 0.52% (3/569) (Fig. 1).

Tick control was reported at all collection locations; however, ticks were found at all the sampled properties. A total of 262 ticks were collected, which were identified as follows: *Dermacentor nitens*, 202; *Amblyomma sculptum*, 61; and *Rhipicephalus (Boophilus) microplus*, 23. Only the ticks of the *A. sculptum* species were subjected to PCR for the detection of *Rickettsia* spp.; however, all the reactions were negative.

Amplification of *A. phagocytophilum* DNA was not observed in the assessed animals. Amplification for GAPDH was noted in all the negative samples.

## Discussion

This study involved the largest number of equids so far for the detection of positivity for antigens of *Rickettsia* spp., *Leishmania* spp., and *Anaplasma* spp. in Brazil and the second largest sample for that of *Borrelia* spp. The study was conducted in a region characterized by a humid tropical climate, favoring the biological cycle of the vectors throughout the year. It is also in the Atlantic Forest biome, where there are various rodents of Brazilian fauna, such as the capybara (*Hydrochaeris hydrochaeris*), which is suspected to play an important role in the life cycles of *Rickettsia* spp. [47] and *Borrelia* spp. [48]. Ticks were observed on all the sampled properties, justifying the ample spread of these pathogens.

The frequency of animals showing antigens related only to *R*. *rickettsii* (14.6%) or *R.* *parkeri* (15.1%) were higher in the areas included in this study than in a neighboring area (5.8% and 8.7%, respectively) [49]. This fact can be explained by the small sample size when compared to our study, which covered the geographical area and a number of samples about ten times larger than those of the neighboring area. Ticks tested positive for *R. parkeri* in this region [49]; therefore, it is possible that animals with elevated anti-*R. parkeri* titers are in fact positive for this pathological agent*.*

Epidemiological surveys carried out on equids with respect to *Rickettsia* spp. rarely assessed risk factors. In the present study, however, the age range below 3 years was identified as a possible risk factor for infection by this pathological agent. This indicates early exposure of equids to the agent, with a possible decrease in circulating antibodies with time. Unfortunately, it was not possible to find data from other authors to promote discussion of the theme.

The results of the present study showed a lower frequency of *Rickettsia* spp. in donkeys and mules than in horses, which corroborates previous findings [50, 51]. This result may be associated with a greater resistance against *Amblyomma* spp. infestation in mules and donkeys than in horses [52]; however, positivity can be high in animals greatly challenged by vectors [53]. Another interesting point was the absence of positivity for *R. rickettsii* antigens in donkeys. This may suggest that besides resistance to vectors, there may be differences in resistance against species of *Rickettsia*.

The absence of molecular detection of the *gltA* gene corroborates the findings of the state of Goiás [54] and those of Bahia [49], where not a single tick collected from horses tested positive for the *gltA* gene.

In the state of Bahia, between 2005 and 2016, there was at least one notification of infections caused by *Rickettsia* spp. per year in human beings, with no deaths [55]. The importance of domestic animals as amplifying hosts of *Rickettsia spp.* is yet to be completely explained [51]. These previously reported findings, together with the frequency observed in the present study, demonstrate that *Rickettsia* spp. is circulating in the state, considering that equids are indicated as sentinel animals by epidemiological studies and that they can be infected without presenting clinical symptomatology [56–58]. Furthermore, the positivity in animals of urban origin, as found in the present study, raises the risk of urbanization of the disease [59].

Borreliosis is a disease that is neglected in most of the world [48]. The few studies on the disease in equids in Brazil indicate distinct seropositivity varying from 7.2 to 44.7% [3, 18, 60–62]. Even in studies carried out in the same state, large variations can occur; for example, among the studies carried out in the state of Pará, a seropositivity of 26.7% [3] was detected in one study while that of 7.2% was detected in another study [18]. The frequency found in the present study was much higher than that found in dogs (1%) from the same microregion [63]. One of the explanations may be the preference of *A. sculptum *(*Amblyomma cajennense* complex) [64] to parasitize equids, making these species better indicators of the presence of the pathological agent than dogs. *Rhipicephalus microplus* tick parasitism may be another important factor because of the possibility of cross-reaction with *Borrelia theileri*, a tick-borne spirochete known to infect cattle and other mammals like horses, sheep, and deer [65].

Similarities in the results of urban and rural animals may reflect the increase in the urban population of vectors (ticks) and reservoirs (capybaras) and demonstrate the risk of human exposition to the pathogen in the urban zone. No previous studies on this type of population were found.

Factors associated with *Borrelia* spp. infection were rarely cited [3, 8, 9, 18, 66], with contact and intensity of tick infestation [61, 67] and age [68] being the most common. In the present study, all the animals presented tick infestation; as such, this variable cannot be evaluated. However, the male sex was identified as a possible protective factor, which may be explained by the fact that most of the male equids/stallions receive differentiated management, which probably leads to lower infestation and exposure to transmitting agents. Following the logic that tick infestation intensity increases the risk of infection, researchers attribute a greater prevalence in a determined location and breed to the environment where they are found, which in reality is more conducive to maintenance of the vector [69].

As in the case of rickettsiosis, horses can be considered sentinels for borreliosis [70], as corroborated by the results of the present study. The detection of *Borrelia* spp. DNA in 43% of the ticks collected from horses [71] enables us to reach the conclusion that equids can be considered reservoirs of infection. Thus, the equids may be a multiplier and spreader of infected vectors to the peri-urban environment, as with *Rickettsia* spp. [59].

The low frequency of *Leishmania* spp. infection in the equids of the present study partly corroborates the data from the Ministry of Health’s National Disease Notification System, which, in 2015, registered only ten cases of human tegumentary leishmaniosis in the municipalities included in the study. There were no recorded cases of visceral leishmaniosis in the region [72], which has approximately 279,464 inhabitants [73].

It can be observed that despite its low frequency in the animals, this rate was much higher than the recorded cases of the disease in humans in the region. This result indicates that equids can also be considered sentinels for leishmaniosis [13], as well as sources of infection for the vectors [74, 75], because DNA of the parasite has already been detected in the blood of horses [15, 22].

Initially, leishmaniosis was described in rural areas because of the characteristics of its vectors, which maintain a sylvatic cycle [76]. Currently, the disease can be identified in urban or peri-urban areas as a result of adaptation of the vector to this new environment and establishment of new reservoirs [15, 21, 22]. Unlike that of *Borrelia* spp. and *Rickettsia* spp., detection of *Leishmania* spp. in animals the present study occurred only in the rural environment. This may be indicative of absence or low prevalence of the vector or the agent from the studied urban environment or of the fact that equids are not good sentinels for this agent in areas with low prevalence of this pathological agent.

Despite the small number of animals that tested positive for *Leishmania* spp., which compromised the performance of statistical analysis, greater positivity was found in mules and donkeys than in horses. Researchers have identified donkeys as probable sources of infection introduction in a Venezuelan outbreak area, because the first skin lesions suggestive of the disease were reported in donkeys coming from the endemic region [74]. Furthermore, it was observed that these animals were the main source of food for phlebotominae; when compared to the harassment of vectors between donkeys, humans, and dogs these animals were observed to be the main food sources for phlebotominae, unlike the vector hosting among donkeys, humans, and dogs [74]. Nevertheless, epidemiological studies showing donkeys and mules as risk factors for leishmaniosis were not found in the literature.

The negative result of the PCR made it impossible to identify the species involved. However, given the absence of recorded cases of visceral leishmaniosis in humans and dogs in the region, it is probable that the evaluated equids are asymptomatic carriers of tegumentary leishmaniosis. This reinforces the importance of controlling the transportation of equids, especially donkeys, from endemic to non-endemic areas.

Despite having evaluated the largest sample of horses in Brazil, it was not possible to detect *A. phagocytophilum* DNA in the animals in the present study. The lack of detection, as well as the detection of low prevalences, as in the findings from eastern Europe (1.4% prevalence) [77], can be explained through the parasitemia of the pathological agent in horses, occurring for a short period (± 129 days) [78, 79]. This makes serology, the most commonly used method [20, 80], more sensitive than PCR. Performing serology would enable identification of chronic cases, confirmation of endemics of the region, and provision of conditions for evaluating the role of equids in the epidemiology of the disease; however, it was not possible to carry it out.

Few reports on co-infections among zoonotic organisms are available in the literature [81, 82]. The present study detected co-infections of *Leishmania* spp., *Borrelia* spp., and *Rickettsia* spp.; to our knowledge, this is the first report of this type of co-infection. It was not possible to determine how these interactions interfere with animal well-being and public health.

## Conclusions

We were able to verify that infections caused by *Leishmania* spp*.*, *Borrelia* spp., and *Rickettsia* spp. are present in the equine population of the studied area with positivity levels above those observed in other species. This may be a good indicator of the species as a sentinel for these infections. Despite the detected frequencies not being considered high, the participation of equids in the transmission cycle of these pathogens to their vectors or to the human population cannot be ruled out. The detection of animals that tested positive for *Borrelia* spp. and *Rickettsia* spp. in the evaluated urban zone is worth highlighting, which demonstrates the risk of these agents for public health in the region.

## Supplementary Information

**Additional file 1: Figure S1.** Geographical indication of the municipalities participating in the study. A (Santa Cruz da Vitória); B (Floresta Azul); C (Ibicaraí); D (Itabuna); E (Itaju do Colônia), F (Itapé). **Table S1.** Distribution of the antibody titers observed against *R. rickettsii* and *R. parkeri* antigens in isolated responses in equids of the Ilhéus-Itabuna microregion, Bahia. **Table S2.** Distribution of infections by *Rickettsia* spp, *Borrelia* spp., and *Leishmania* spp. in naturally infected equids, according to municipality in the Ilhéus-Itabuna microregion, Bahia. **Table S3**. Generalized bivariate linear models for factors associated with *Rickettsia* spp. infection in naturally infected equids from the Ilhéus-Itabuna microregion, Bahia. **Table S4.** Generalized linear classic multivariate model for factors associated with *Rickettsia* spp. infection in naturally infected equids from the Ilhéus-Itabuna microregion, Bahia. Full model. **Table S5**. Generalized bivariate linear models for factors associated with *Borrelia* spp. infection in naturally infected equids from the Ilhéus-Itabuna microregion, in the state of Bahia. **Table S6.** Generalized linear mixed multivariate model for factors associated with *Borrelia* spp. infection in naturally infected equids from the Ilhéus-Itabuna microregion, Bahia. Full model. **Table S7**. Generalized bivariate linear models for factors associated with *Leishmania* spp. infection in naturally infected equids from the Ilhéus-Itabuna microregion, Bahia.

## Data Availability

http://www.biblioteca.uesc.br/biblioteca/bdtd/201370049T.pdf. Other data will be made available on request.
