# Supplementary material for: Frequency and factors associated of potential zoonotic pathogens (Borrelia spp., Rickettsia spp., Leishmania spp., and Anaplasma phagocytophilum) in equids in the state of Bahia, Brazil
Source: Parasit Vectors. 2021 May 22;14:275. doi: 10.1186/s13071-021-04777-4 (PMC8140576; doi:10.1186/s13071-021-04777-4)
Supplement: Supplementary file 1 — Additional file 1: Figure S1. Geographical indication of the municipalities participating in the study. A (Santa Cruz da Vitória); B (Floresta Azul); C (Ibicaraí); D (Itabuna); E (Itaju do Colônia), F (Itapé). Table S1. Distribution of the antibody titers observed against R. rickettsii and R. parkeri antigens in isolated responses in equids of the Ilhéus-Itabuna microregion, Bahia. Table S2. Distribution of infections by Rickettsia spp, Borrelia spp., and Leishmania spp. in naturally infected equids, according to municipality in the Ilhéus-Itabuna microregion, Bahia. Table S3. Generalized bivariate linear models for factors associated with Rickettsia spp. infection in naturally infected equids from the Ilhéus-Itabuna microregion, Bahia. Table S4. Generalized linear classic multivariate model for factors associated with Rickettsia spp. infection in naturally infected equids from the Ilhéus-Itabuna microregion, Bahia. Full model. Table S5. Generalized bivariate linear models for factors associated with Borrelia spp. infection in naturally infected equids from the Ilhéus-Itabuna microregion, in the state of Bahia. Table S6. Generalized linear mixed multivariate model for factors associated with Borrelia spp. infection in naturally infected equids from the Ilhéus-Itabuna microregion, Bahia. Full model. Table S7. Generalized bivariate linear models for factors associated with Leishmania spp. infection in naturally infected equids from the Ilhéus-Itabuna microregion, Bahia. [file 13071_2021_4777_MOESM1_ESM.docx]

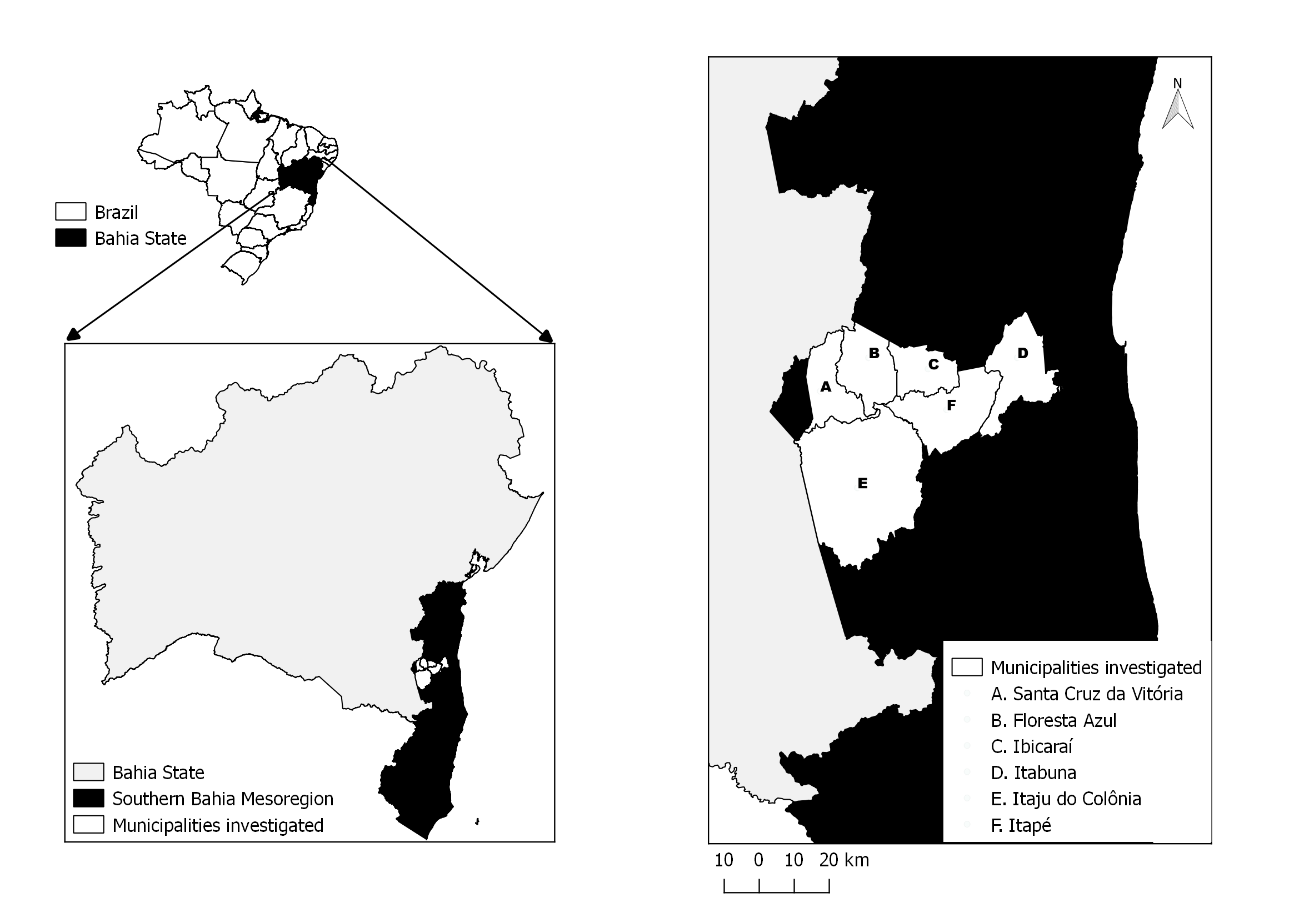


**Figure S1.**

**Table S1.**

| **Titers** | **Reactive animals only for *Rickettsia rickettsii*** | **Reactive animals only for *Rickettsia parkeri*** |
| --- | --- | --- |
| 1:64 | 57 (68,7%) | 70 (81,4%) |
| 1:128 | 19 (22,9%) | 11 (12,8%) |
| 1:256 | 4 (4,8%) | 3 (3,5%) |
| 1:516 | 1 (1,2%) | 2 (2,3%) |
| 1:1024 | 2 (2,4%) | - |
| TOTAL | 83 (100 %) | 86 (100%) |

**Table S2.**

| **Municipalities** | **Reactive animals for *Rickettsia* spp.** | **Reactive**  **animals for *Borrelia* spp.** | **Reactive**  **animals for *Leishmania* spp.** |
| --- | --- | --- | --- |
| **Floresta Azul** | 33 (17,5%) | 16 (20,2%) | 2 (9,5%) |
| **Ibicaraí** | 28 (14,8%) | 5 (6,3%) | 3 (14%) |
| **Itajú do Colônia** | 76 (40,4%) | 21 (26,5%) | 7 (33,3%) |
| **Itapé** | 15 (7,9%) | 14 (17,7%) | 2 (9,5%) |
| **Santa Cruz da Vitória** | 24 (12,7%) | 16 (20,2%) | 6 (28,5%) |
| **Itabuna** | 12 (6,3%) | 7 (8,8%) | 0 (0%) |
| **Total** | 188 (100%) | 79 (100%) | 21 (100%) |

**Table S3**.

| **Variable** | **Equidae** | | | | **Odds ratio** | ***P*** |
| --- | --- | --- | --- | --- | --- | --- |
|  | **Positive (%)** | | **Negative (%)** | | **(95% CI)** |  |
| **Sex** |  |  |  |  |  |  |
| Male | 51 | 36.2 | 90 | 63.8 | 1.13 (0.76-1.70) | 0.55 |
| Female (Ref) | 125 | 33.3 | 250 | 66.7 |  |  |
| **Species** |  |  |  |  |  |  |
| Horses (Ref) | 169 | 35.6 | 306 | 64.4 |  |  |
| Donkey or mule | 07 | 17.1 | 34 | 82.9 | 0.37 (0.16-0.86) | 0.02 |
| **Animals kept in stables** |  |  |  |  |  |  |
| Yes (ref) | 16 | 33.3 | 32 | 66.7 |  |  |
| No | 160 | 34.2 | 308 | 65.8 | 1.04 (0.55-1.95) | 0.90 |
| **Contact with cattle** |  |  |  |  |  |  |
| Yes (Ref) | 144 | 32.9 | 293 | 67.1 |  |  |
| No | 32 | 40.5 | 47 | 59.5 | 1.39 (0.85-2.26) | 0.20 |
| **Age range** |  |  |  |  |  |  |
| Young | 41 | 47.7 | 45 | 52.3 | 2.05 (1.21-3.49) | <0.01 |
| Adult | 80 | 31.9 | 171 | 68.1 | 1.05 (0.70-1.60) | 0.80 |
| Senior (Ref) | 55 | 30.7 | 124 | 69.3 |  |  |

**Table S4.**

| **Variable** | **Category** | **Odds ratio**  **(95%CI)** | ***P*** |
| --- | --- | --- | --- |
| **Species** | Horses (Ref) |  |  |
|  | Donkey or mule | 0.40 (0.17-0.94) | 0.03 |
| **Contact with cattle** | Yes (Ref) |  |  |
|  | No | 1.36(0.81-2.29) | 0.25 |
| **Age range** | Young | 2.13 (1.22-3.71) | 0.01 |
|  | Adult | 1.11 (0.73-1.70) | 0.61 |
|  | Senior (Ref) |  |  |
| **Animals kept in stables** | Yes (Ref) |  |  |
|  | No | 1.34(0.69-2.60) | 0.38 |
| **Sex** | Male | 1.07(0.69-1.66) | 0.77 |
|  | Female (Ref) |  |  |

AIC=660.3 (Akaike Information Criterion)

**Table S5**.

| **Variable** | **Equidae** | | | | **Odds ratio** | ***P*** |
| --- | --- | --- | --- | --- | --- | --- |
|  | **Positive %** | | **Negative %** | | **(CI 95%)** |  |
| **Sex** | | |  |  |  |  |
| Male | 10 | (7,1) | 131 | (92,9) | 0,42 (0,19-0,91) | 0,03 |
| Female (Ref) | 62 | (16,5) | 313 | (83,5) |  |  |
| **Contact with cattle** | | |  |  |  |  |
| Yes | 59 | (13,5) | 378 | (86,5) | 0,71 (0,24-2,10) | 0,53 |
| No (Ref) | 13 | (16,5) | 66 | (83,5) |  |  |
| **Contact with goats** | | |  |  |  |  |
| Yes | 7 | (11,7) | 53 | (88,3) | 0,92 (0,21-3,97) | 0,91 |
| No (Ref) | 65 | (14,3) | 391 | (85,7) |  |  |
| **Contact with poultry** | | |  |  |  |  |
| Yes | 68 | (15,2) | 379 | (84,8) | 2,79 (0,81-9,58) | 0,10 |
| No (Ref) | 4 | (5,8) | 65 | (94,2) |  |  |
| **Contact with sheep** | | |  |  |  |  |
| Sim | 16 | (15,1) | 90 | (84,9) | 1,18 (0,44-3,18) | 0,75 |
| Não (Ref) | 56 | (13,7) | 354 | (86,3) |  |  |
| **Animals kept in stables** | | | |  |  |  |
| Yes (Ref) | 6 | (12,5) | 42 | (87,5) |  |  |
| No | 66 | (14,1) | 402 | (85,9) | 0,92 (0,34-2,48) | 0,87 |
| **Uses antiparasitic on the equine** | | | | |  |  |
| Yes (Ref) | 58 | (12,6) | 403 | (87,4) |  |  |
| No | 14 | (25,4) | 41 | (74,5) | 2,60 (0,76-8,93) | 0,13 |
| **Species** | | |  |  |  |  |
| Horse (Ref) | 64 | (13,5) | 411 | (86,5) |  |  |
| Donkey or mule | 8 | (19,5) | 33 | (80,5) | 1,24 (0,47-3,23) | 0,66 |
| **Toxic plants in the farm** | | | | |  |  |
| Yes | 33 | (14,3) | 198 | (85,7) | 1,17 (0,53-2,57) | 0,71 |
| No (Ref) | 39 | (13,7) | 246 | (86,3) |  |  |
| **Rats in the farm** | | |  |  |  |  |
| Yes | 63 | (15,3) | 348 | (84,7) | 1,70 (0,67-4,34) | 0,27 |
| No (Ref) | 9 | (8,6) | 96 | (91,4) |  |  |
| **Age range** | | | |  |  |  |
| Young (Ref) | 13 | (15,1) | 73 | (84,9) |  |  |
| Adult | 32 | (12,7) | 219 | (87,3) | 0,80 (0,39-1,66) | 0,56 |
| Senior | 27 | (15,1) | 152 | (84,9) | 0,96 (0,45-2,05) | 0,91 |

**Table S6.**

| **Variable** | **Category** | **Odds ratio (95%CI)** | ***P*** |
| --- | --- | --- | --- |
| Age range | Young (Ref) |  |  |
|  | Adult | 0,69 (0,33-1,46) | 0,33 |
|  | Senior | 0,80 (0,36-1,74) | 0,57 |
| Species | Horse (Ref) |  |  |
|  | Donkey or mule | 1,28 (0,49-3,31) | 0,62 |
| Animals kept in stables | Yes (Ref) |  |  |
|  | No | 0,67 (0,25-1,84) | 0,44 |
| Contact with cattle | No (Ref) |  |  |
|  | Yes | 1,41 (0,57-3,49) | 0,46 |
| Contact with goats | No (Ref) |  |  |
|  | Yes | 0,26 (0,06-1,14) | 0,07 |
| Contact with poultry | No (Ref) |  |  |
|  | Yes | 1,66 (0,49-5,58) | 0,42 |
| Contact with sheep | No (Ref) |  |  |
|  | Yes | 1,66 (0,64-4,33) | 0,30 |
| Rats in the farm | No (Ref) |  |  |
|  | Yes | 1,65 (0,70-3,88) | 0,25 |
| Sex | Female (Ref) |  |  |
|  | Male | 0,34 (0,15-0,78) | 0,01 |
| Toxic plants in farm | No (Ref) |  |  |
|  | Yes | 1,50 (0,72-3,13) | 0,28 |

AIC=425,1 (Akaike Information Criterion)

**Table S7**.

| **Variable** | **Equidae** | | | | **Odds ratio** | ***P*** |
| --- | --- | --- | --- | --- | --- | --- |
|  | **Positive %** | | **Negative %** | | **CI 95%** |  |
| **Species** | |  |  |  |  |  |
| Horse (Ref) | 14 | (2,9) | 461 | (97,0) |  |  |
| Donkey or mule | 6 | (14,6) | 35 | (85,3) | 5,64 (2,04-15,59) | < 0,01 |
| **Sex** |  |  |  |  |  |  |
| Male | 3 | (2,1) | 138 | (97,9) | 0,45 (0,13 -1,58) | 0,21 |
| Female (Ref) | 17 | (4,5) | 358 | (95,5) |  |  |
| **Animal kept in stables** | |  |  |  |  |  |
| Yes (Ref) | 2 | (4,2) | 46 | (95,8) |  |  |
| No | 18 | (3,9) | 450 | (96,1) | 0,92 (0,20-4,09) | 0,91 |
| **Toxic plants in the farm** | |  |  |  |  |  |
| Yes | 9 | (3,9) | 222 | (96,1) | 1,00 (0,41-2,4) | 0,98 |
| No (Ref) | 11 | (3,8) | 274 | (96,1) |  |  |
| **Contact with cattle** | |  |  |  |  |  |
| Yes (Ref) | 19 | (4,4) | 418 | (95,7) |  |  |
| No | 1 | (1,3) | 78 | (98,7) | 0,28 (0,03-2,13) | 0,22 |
| **Contact with poultry** |  |  |  |  |  |  |
| Yes (Ref) | 20 | (4,5) | 427 | (95,5) |  |  |
| No | 0 | (0) | 69 | (100) | Undefined | 0,99 |
| **Contact with goats** | |  |  |  |  |  |
| Yes (Ref) | 6 | (10) | 54 | (90) |  |  |
| No | 14 | (3,1) | 442 | (97) | 0,28 (0,10-0,77) | 0,01 |
| **Stocks food in the farm** | |  |  |  |  |  |
| Yes | 13 | (3,3) | 385 | (96,7) | 0,53 (0,20 -1,37) | 0,19 |
| No (Ref) | 7 | (5,9) | 111 | (94,1) |  |  |
| **Contact with sheep** |  |  |  |  |  |  |
| Yes (Ref) | 9 | (8,49) | 97 | (91,5) |  |  |
| No | 11 | (2,7) | 399 | (97,3) | 0,29 (0,11-0,73) | < 0,01 |
| **Rats in the farm** |  |  |  |  |  |  |
| Yes | 20 | (4,9) | 391 | (95,1) | Undefined | 0,98 |
| No (Ref) | 0 | (0) | 105 | (100) |  |  |
| **Contact with cats** |  |  |  |  |  |  |
| Yes | 18 | (4,4) | 390 | (95,6) | 2,44 (0,55 – 10,70) | 0,23 |
| No (Ref) | 2 | (1,9) | 106 | (98,2) |  |  |
| **Age range** | |  |  |  |  |  |
| Young (Ref) | 1 | (1,2) | 85 | (98,8) |  |  |
| Adult | 10 | (4,0) | 241 | (96,0) | 3,5 (0,44-27,93) | 0,23 |
| Senior | 9 | (5,0) | 170 | (95,0) | 4,49 (1,5-36,07) | 0,15 |
| **Municipalities** | |  |  |  |  |  |
| Itapé | 2 | (3,0) | 65 | (97,0) | 0,78 (0,15-3,8) | 0,76 |
| Itaju (Ref) | 7 | (3,8) | 179 | (96,2) |  |  |
| Santa Cruz | 6 | (7,1) | 79 | (92,9) | 1,94 (0,63-5,96) | 0,24 |
| Ibicarai | 3 | (3,8) | 77 | (96,3) | 0,99 (0,25-3,95) | 0,99 |
| Floresta Azul | 2 | (2,0) | 96 | (98,0) | 0,53 (0,10-2,61) | 0,43 |
